# New Insulating Antiferromagnetic Quaternary Iridates *M*La_10_Ir_4_O_24_ (*M* = Sr, Ba)

**DOI:** 10.1038/srep11705

**Published:** 2015-07-01

**Authors:** Qingbiao Zhao, Fei Han, Constantinos C. Stoumpos, Tian-Heng Han, Hao Li, J. F. Mitchell

**Affiliations:** 1 Materials Science Division, Argonne National Laboratory, Argonne, Illinois 60439, United States; 2 Department of Physics, University of Chicago, Chicago, IL 60637, USA

## Abstract

Recently, oxides of Ir^4+^ have received renewed attention in the condensed matter physics community, as it has been reported that certain iridates have a strongly spin-orbital coupled (SOC) electronic state, *J*_eff_ = ½, that defines the electronic and magnetic properties. The canonical example is the Ruddlesden-Popper compound Sr_2_IrO_4_, which has been suggested as a potential route to a new class of high temperature superconductor due to the formal analogy between *J*_eff_ = ½ and the *S* = ½ state of the cuprate superconductors. The quest for other iridium oxides that present tests of the underlying SOC physics is underway. In this spirit, here we report the synthesis and physical properties of two new quaternary tetravalent iridates, *M*La_10_Ir_4_O_24_ (*M* = Sr, Ba). The crystal structure of both compounds features isolated IrO_6_ octahedra in which the electronic configuration of Ir is d^5^. Both compounds order antiferromagnetically despite the lack of obvious superexchange pathways, and resistivity measurement shows that SrLa_10_Ir_4_O_24_ is an insulator.

Electrons in 3*d* transition metal oxides exhibit correlated behavior because the bandwidth, *W*, is relatively narrow, while the electron-electron repulsion, parametrized by a Hubbard *U*, is significant. The result is a set of collective phenomena including high temperature superconductivity^1^ and colossal magnetoresistance^2^,^3^. On the other hand, 5*d* transition metals are characterized by more extended orbitals, and in general, are expected to be uncorrelated metals, as for example IrO_2_^4^ and Bi_2_Ir_2_O_7_^5^. Recently, however, Kim *et al.* found that strong spin-orbital coupling in the layered compound Sr_2_IrO_4_ leads to a relatively narrow *J*_eff_ = ½ band whose width is of the same scale as electron correlation, yielding a Mott insulating state^6^,^7^. With a square IrO_2_ network, a *U/W *~ 1, and a magnon dispersion qualitatively the same as cuprates^8^, Sr_2_IrO_4_ has been studied intensely as a potential route to a new class of high temperature superconductor^8^,^9^,^10^. Some known iridates, including Ba_2_IrO_4_^11^ and Ca_4_IrO_6_^12^, have already shown behavior consistent with a *J*_eff_ = ½ description by resonant inelastic X-ray scattering, indicating that the phase space of *J*_eff_ = ½ materials extends beyond Sr_2_IrO_4_. These discoveries underscore the importance of identifying new iridates with *J*_eff_ = ½ states to better understand the phenomenology of these unusual correlated oxides.

The scope of the present work lies firmly in the regime of discovery synthesis of new compounds in a relatively unexplored regime of crystal chemistry as a first step on the way to classifying and understanding the breadth of spin-orbit driven physics in iridates. Toward this end, we have synthesized two new isostructural tetravalent iridates, *M*La_10_Ir_4_O_24_ (*M* = Sr, Ba) and characterized their crystal structures and magnetic, transport, and thermodynamic signatures. Each is comprised of isolated IrO_6_ octahedra in which the nominal electronic configuration of Ir is d^5^. They both order antiferromagnetically, and resistivity measurement shows that SrLa_10_Ir_4_O_24_ exhibits insulating behavior.

The extensively studied iridates are mostly ternary oxides, which is likely due to the lack of approaches for crystallizing iridates that are compositionally diverse. To expand the horizon in studying iridates as *J*_eff_ = 1/2 candidates, quaternary and even higher order iridates are desired. Here it is demonstrated that high order iridates can be made with a facile flux crystal growth approach.

## Results

**Figure 1:**
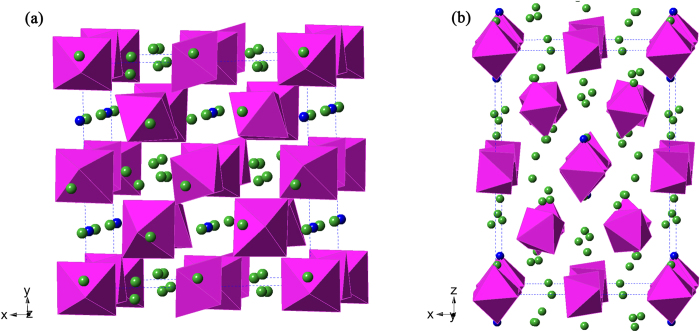
The crystal structure of *M*La_10_Ir_4_O_24_ (*M* = Sr, Ba). The purple color denotes IrO_6_ octahedra, and the green and blue spheres denote La and *M* atoms (*M* = Sr, Ba), respectively (**a**) viewed from approximately (001) direction (**b**) viewed approximately from (010) direction.

Lattice constants and space group (*I*4_*1*_*/a*) extracted from single-crystal diffraction measurements were similar to that of the known compound Sr_9_La_2_Mo_4_O_24_^13^, and indeed the crystal structures (see Fig. 1) of the iridates could be solved from this starting model. For SrLa_10_Ir_4_O_24_, no discernible site mixing between Sr and La was found in the refinement. For BaLa_10_Ir_4_O_24_, the Ba and La sites were assigned based on the apparent differences in bond lengths of Ba-O and La-O. The structure features isolated IrO_6_ octahedra with *M* (*M* = Sr, Ba) and La atoms located interstitially. There are two independent Ir sites, both occupying special positions, with the Ir atoms in a distorted octahedral coordination environment. The lattice parameters of SrLa_10_Ir_4_O_24_ are *a* = 11.58 Å, *c* = 16.24 Å, while those of BaLa_10_Ir_4_O_24_ are *a* = 11.66 Å, *c* = 16.17 Å. Compared to SrLa_10_Ir_4_O_24_, the Ba analogue has a larger *a* lattice parameter and a smaller *c* lattice parameter. In other words, the lattice is “squashed” rather than simply enlarged. The cell volume of BaLa_10_Ir_4_O_24_ (2196.9 Å^3^) is only slightly larger than that of SrLa_10_Ir_4_O_24_ (2183.4 Å^3^), a difference of ~0.05%. This weak volume perturbation is not surprising, because although the ionic radius of Ba^2+^ (1.42 Å) is significantly larger than that of Sr^2+^ (1.26 Å), the alkaline earth metal is only a small fraction of the unit cell contents. For SrLa_10_Ir_4_O_24_, the bond length of Ir1-O ranges from 1.967(15) Å to 2.030(16) Å, while the bond length of Ir2-O ranges from 2.001(16) Å to 2.073(16) Å. For BaLa_10_Ir_4_O_24_, the bond length of Ir1-O ranges from 1.983(12) Å to 2.046(12) Å, while the bond length of Ir2-O ranges from 2.006(12) Å to 2.086(13) Å. These bond lengths are typical of Ir(IV) in oxides^14^,^15^.

**Figure 2:**
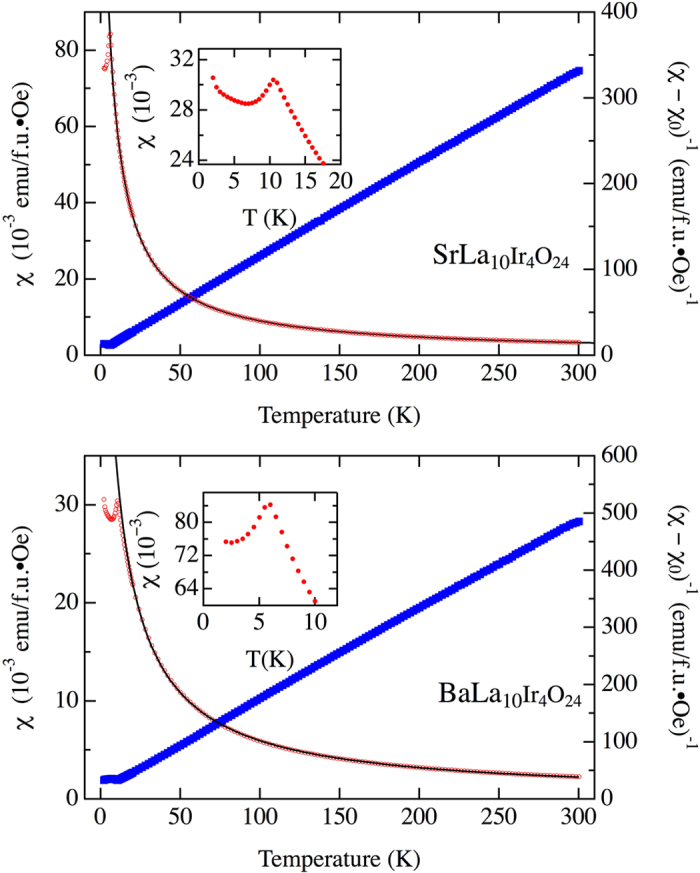
Temperature dependent DC magnetic susceptibility of SrLa_10_Ir_4_O_24_ (top) and Ba La_10_Ir_4_O_24_ (bottom). Open red circles are the data, and the solid black line is a Curie-Weiss fit including a temperature independent parameter, χ_0_, which takes the values 2.2 × 10^−4^ emu/f.u.•Oe and 3.7 × 10^−4^ emu/f.u•Oe, respectively. The blue line is the inverse T-dependent component of the susceptibility. Measuring field is 5000 Oe. The insets show the low temperature ranges (**a**) SrLa_10_Ir_4_O_24_ (**b**) BaLa_10_Ir_4_O_24_.

The temperature-dependent magnetic susceptibility of SrLa_10_Ir_4_O_24_ shows a cusp characteristic of antiferromagnetic order at T_N_ = 12 K (Fig. 2, top), indicating a non-negligible magnetic exchange among the isolated octahedra. Above this cusp, the magnetic behavior is well approximated as that of a Curie-Weiss paramagnet. A fit of the data to χ = C/(T – θ) + χ_0_ (where χ_0_ phenomenologically accounts for all diamagnetic contributions) from 20 K to 300 K (the results are largely insensitive to the choice of fitting range) gives a Weiss constant of −8.5 K, in good agreement with T_N_. The effective moment (μ_eff_) of 1.11 μ_B_/Ir is considerably reduced from the 1.73 μ_B_/Ir expected for low-spin d^5^ Ir(IV) in a rigorously *J*_eff_ = ½ configuration (cubic crystal field, k_B_T/**λ** **→** 0)^16^. We note that such reduced effective moments are not uncommonly reported among iridates, including Sr_2_IrO_4_ (0.50 μ_B_ /Ir)^17^, Sr_3_Ir_2_O_7_ (0.69 μ_B_ /Ir)^18^, 9 M BaIrO_3_ (0.13 μ_B_ /Ir)^17^ and 6 M BaIrO_3_ (0.276 μ_B_ /Ir)^19^. However, other iridates, notably Na_2_IrO_3_^20^, with a reported μ_eff_ = 1.79 μ_B_ /Ir, follow more closely the expected behavior. Discrepancies such as these suggest an ‘effective *g*-factor’ significantly reduced from the free electron value, deriving potentially from non-cubic symmetry, an admixture of configurations other than t_2g_^5^, or the effect of hybridization with the O sublattice network.

Similarly, the temperature dependent magnetic measurement of BaLa_10_Ir_4_O_24_ shows antiferromagnetic ordering at a somewhat lower temperature, T_N_ = 6 K (Fig. 2, bottom), and also follows Curie-Weiss behavior above this temperature, with μ_eff_ = 1.35 μ_B_, slightly larger than that of SrLa_10_Ir_4_O_24_. The Weiss constant obtained is −5.1 K, close to the measured T_N_. For both SrLa_10_Ir_4_O_24_ and BaLa_10_Ir_4_O_24_, at temperatures below the T_N_, the susceptibility has a small upturn, which may arise from a small amount of paramagnetic impurity spins.

**Figure 3:**
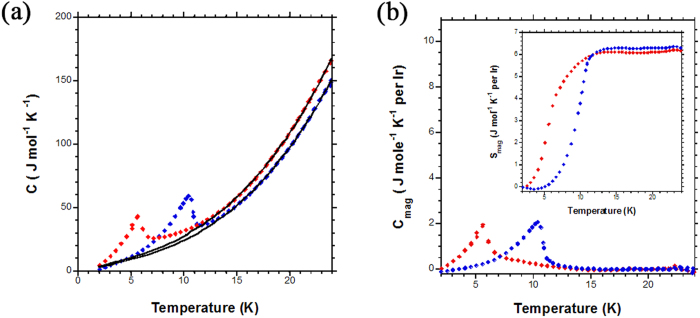
Heat capacity of SrLa_10_Ir_4_O_24_ and BaLa_10_Ir_4_O_24_. Red points are for SrLa_10_Ir_4_O_24_ and blue points are for BaLa_10_Ir_4_O_24_. Fits are shown in black (see text). (**a**) Total heat capacity (**b**) magnetic contribution to the heat capacity. Inset to (**b**) shows the entropy of the magnetic transition.

Heat capacity measurements were carried out on both compounds. Above the magnetic transition, the data can be described by the expression C = C_electron_ + C_phonon_ = γT+ βT^3^ (Fig. 3a). The values of γ and β extracted from the fits for SrLa_10_Ir_4_O_24_ are 0.405 Jmol^−1^K^−2^ and 0.00205 Jmol^−1^K^−4^, and for BaLa_10_Ir_4_O_24_ γ and β value are 0.47 Jmol^−1^K^−2^ and 0.00224 Jmol^−1^K^−4^. Corresponding Debye temperatures were calculated to be 333 K for SrLa_10_Ir_4_O_24_, and 324 K for BaLa_10_Ir_4_O_24_. The magnetic entropy in the low-T regime can then be calculated as S_mag_(T) = 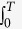 C_mag_/T dT (Fig. 3b), where C_mag_ = C_tot_ – C_electron_ - C_phonon_, yielding S_mag_ = 6.31 J mol^−1^ K^−1^ for SrLa_10_Ir_4_O_24_ and 6.17 J mol^−1^ K^−1^ for BaLa_10_Ir_4_O_24_ (Fig. 3b). The expected entropy of the magnetic transition (S_mag_) equals to Rln(2 J+1), where R is the gas constant, and J is the total angular momentum. For *J*_eff_ = ½, the expected value is 5.76 J mol^−1^ K^−1^, in fair agreement with that measured here.

**Figure 4:**
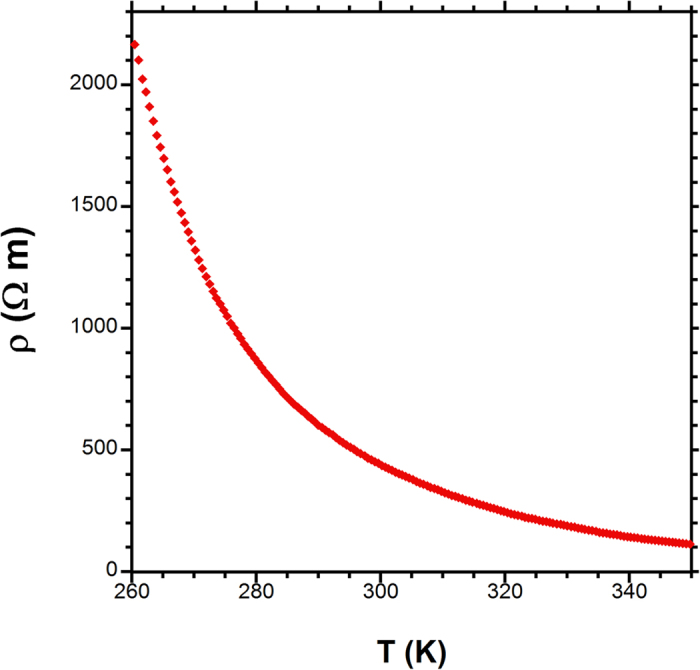
Resistivity vs. temperature for SrLa_10_Ir_4_O_24_.

Resistivity measurement shows that SrLa_10_Ir_4_O_24_ exhibits insulating behavior (Fig. 4), which is expected given that the crystal structure features isolated IrO_6_ octahedra. With the caveat that the behavior is evaluated in a narrow temperature range of 260 K to 350 K, it was found that the resistivity is best modeled by simple thermally activated hopping, with Ea ~0.26 eV, while three-dimensional and two-dimensional variable range hopping and small polaron models yield poorer agreement with the measured data. Unfortunately, BaLa_10_Ir_4_O_24_ crystal specimens are too small for a conductivity measurement at this time. Due to the similar crystal structure, one may expect similar electronic transport behavior to that of the Sr analogue.

## Discussion

Flux crystal growth is an important approach to grow single crystals of new materials^21^,^22^,^23^,^24^,^25^,^26^. For exploratory crystal growth of new iridates, KOH and K_2_CO_3_ fluxes have typically been used^27^,^28^. Remarkably, for the synthesis of SrLa_10_Ir_4_O_24_, Ir metal was used as the source of Ir and was oxidized to Ir(IV) in the SrCl_2_ flux. It is known that some fluxes like KOH can dissolve O_2_ from the atmosphere to provide an oxidizing environment; mostly Ir(V) compounds have been synthesized from KOH flux, but some Ir(VI) and Ir(V) oxides are also reported^29^,^30^. Evidently SrCl_2_ also dissolves sufficient O_2_ from the atmosphere to oxidize Ir metal to Ir(IV). However, under the conditions of our synthesis, SrCl_2_ apparently provides a less oxidizing environment compared to KOH, as we found no higher oxidation state products. EDS shows no evidence of chlorine incorporation in the crystals.

*M*La_10_Ir_4_O_24_ (*M* = Sr, Ba) compounds have similar lattice parameters as the Mo(VI) oxide, Sr_9_La_2_Mo_4_O_24_^13^. Although the atomic coordinates of Sr_9_La_2_Mo_4_O_24_ were not reported^13^, the stoichiometry and similar lattice parameters leads one to expect that the structures of *M*La_10_Ir_4_O_24_ (*M* = Sr, Ba) and Sr_9_La_2_Mo_4_O_24_ are closely related. Apparently this structure type can adjust its *M*:La ratio to accommodate either the tetravalent Ir or the hexavalent Mo. When only alkaline earth metal is involved and rare earth metal is excluded, the structure type can host transition metal with mixed oxidation states, which has been shown by the synthesis of Ca_11_Re_4_O_24_^31^ Sr_11_Re_4_O_24_^32^ and Ba_11_Os_4_O_24_^33^.

Magnetization data show that the effective moment of *M*La_10_Ir_4_O_24_ (*M* = Sr, Ba) is significantly reduced vis-à-vis that expected for a *J* = ½ Kramer’s ion. In the case of 6 M-BaIrO_3_ it has been suggested that such a reduced effective moment may arise from the *d* electron hybridization with oxygen p states^18^. Putting any such argument on a stronger, more quantitative footing calls for a broader materials search and theoretical input beyond the scope of this report.

As mentioned earlier, the *J*_eff_ = ½ state has been implicated as foundational to the understanding of iridate physics, although this description rigorously applies only in the case of an isolated and ideal octahedral crystal field^6^,^7^. The former criterion eliminates band structure effects and super-exchange, while the latter guarantees the symmetry of the *J*_eff_ = ½ wavefunction (assuming that the e_g_ states lie at sufficiently high energy that the contribution from excited configurations such as t_2_ _g_^4^e_g_^1^ are negligible. This latter assumption has been questioned recently by Katakuri *et al.* from quantum chemical calculations^34^. By isolating the octahedra and thus eliminating bandwidth and super-exchange as a competing influence on the electronic structure^35^, compounds such as *M*La_10_Ir_4_O_24_ offer a platform for testing the intrinsic nature of the *J*_eff_ = ½ description.

## Conclusion

In summary, we report the discovery and characterization of two new quaternary iridates, SrLa_10_Ir_4_O_24_ and BaLa_10_Ir_4_O_24_, with the crystal structure similar to Ca_11_Re_4_O_24_^31^, Sr_11_Re_4_O_24_^32^ and Ba_11_Os_4_O_24_^33^ and Sr_9_La_2_Mo_4_O_24_^13^. By isolating the octahedra and thus eliminating bandwidth and super-exchange as a competing influence on the electronic structure, compounds such as these can provide a platform for testing the detailed nature and range of applicability of the *J*_eff_ = ½ description using, for example, resonant inelastic x-ray scattering. More generally, the synthetic approaches reported here provide valuable insights that can stimulate the efforts in crystal growth of new iridates, particularly quaternary iridates, that will be essential to achieving a broader understanding of correlated electron physics in the presence of strong spin-orbit coupling.

During the proofing process of this manuscript, we became aware of a paper reporting the synthesis and magnetic properties of SrxLa11−xIr4O24 (B.F. Phelan *et al.* Phys. Rev. B 91, 155117 (2015)). The crystallographic data presented by Phelan *et al.* for single crystal Sr4.25La6.75Ir4O24 are qualitatively the same as ours with a slightly smaller average Ir-O bond length that can be attributed to a slightly more Ir(V) concentration in the specimen of Phelan *et al.* Magnetic properties of the x = 1 member of this series (polycrystalline specimens in the Phelan *et al.* report) are comparable to those here, showing Curie-Weiss behavior at a cusp at ~12 K., Curiously, the effective moment for SrLa10Ir4O24 reported by Phelan et al differs considerably from that we find and is closer to that expected by an isolated Ir(IV). We do not have an explanation for this discrepancy, but note that the synthetic processes are different.

## Methods

### Syntheses

Crystals of the *M*La_10_Ir_4_O_24_ (*M* = Sr, Ba) were grown by a flux method. For the synthesis of SrLa_10_Ir_4_O_24_, La_2_O_3_ (Alfa Aesar, 99.9%, 0.51 mmol), Ir metal (0.5 mmol), and anhydrous SrCl_2_ (12.6 mmol) were loaded into a platinum crucible. The crucible was placed into a box furnace, heated to 1200 °C at 300 °C/hour, held at that temperature for 12 h, cooled to 900 °C at 12 °C/hour, and finally cooled to room temperature by turning off the furnace. For the synthesis of BaLa_10_Ir_4_O_24_, La_2_O_3_ (Alfa Aesar, 99.9%, 0.82 mmol) IrO_2_ (0.82 mmol), anhydrous BaCl_2_ (30 mmol) were loaded into a platinum crucible. The crucible was placed into a box furnace, heated to 900 °C at 300 °C/hour, then heated to 1200 °C at 12 °C/hour, held at 1200 °C for 12 h, cooled to 950 °C at 10 °C/hour, and finally cooled to room temperature by turning off the furnace. For both compounds, the crystals were separated from the flux by dissolving the flux in water aided by sonication, and then isolated with vacuum filtration and rinsing with acetone. The crystals are stable in air and water. They are black in color with an irregular polyhedral shape, and the crystal sizes are about 100 microns from one face to the face across.

### Single Crystal X-ray Diffraction and EDS

Single crystals with irregular polyhedral shape were selected and mounted on tips of glass fibers for X-ray diffraction. Intensity data were collected at room temperature on a STOE imaging plate diffraction system (IPDS-II) using graphite-monochromatized Mo–Kα radiation (λ = 0.71073 Å) operating at 50 kV and 40 mA with a 34 cm diameter imaging plate. For SrLa_10_Ir_4_O_24_, individual frames were collected with a 15 min exposure time and a 1° ω rotation at a φ angle of 98°, while for BaLa_10_Ir_4_O_24_, individual frames were collected with a 5 min exposure time and a 1° ω rotation at a φ angle of 78°. Data reduction and integration absorption correction were performed using X-Area software provided by STOE, and the crystal structures were solved with SHELXL 97 software package^36^. The parameters for data collection and the details of the structure refinement are given in Table 1. Atomic coordinates, isotropic thermal displacement parameters (*U*_*eq*_) and occupancies of all atoms are given in Table 2, and selected bond lengths are given in Tables 3 for both compounds. Anisotropic displacement parameters are given in the supplemental material. The isotropic thermal parameter for Sr1 in SrLa_10_Ir_4_O_24_ is relatively large, and the anisotropic thermal parameters for Sr1 have an elongated ellipsoid shape. This may be a sign of disordering for this site. The Ba in BaLa_10_Ir_4_O_24_, on the other hand, is well behaved. Electron dispersive X-ray spectroscopy data were collected on Oxford INCA Model 6498 and no discernible chlorine peaks were detected.

### Magnetism

The DC magnetic susceptibilities of the ground samples were measured using a Quantum Design MPMS XL SQUID magnetometer. Samples were measured under zero-field-cooled (ZFC) and field-cooled (FC) conditions in an applied field of 5000 G. For SrLa_10_Ir_4_O_24_, the magnetization was measured upon warming the samples from 1.8 to 300 K. For BaLa_10_Ir_4_O_24_, the magnetization was measured upon warming the samples from 2 to 300 K. The very small diamagnetic contribution of the gelatin capsule had a negligible contribution to the overall magnetization and was not subtracted.

### Electrical Conductivity and Heat Capacity

Electrical conductivity of a single crystal of SrLa_10_Ir_4_O_24_ was measured on a Quantum Design PPMS with a four-probe method. It was found that below 260 K the resistance is too large to be measured, thus data between 260 K and 350 K were measured. Heat capacity for both compounds was measured on the PPMS from 2 K to 30 K.

## Additional Information

**How to cite this article**: Zhao, Q. *et al.* New Insulating Antiferromagnetic Quaternary Iridates MLa10Ir4O24 (M = Sr, Ba). *Sci. Rep.* **5**, 11705; doi: 10.1038/srep11705 (2015).

## Supplementary Material

Supplementary Information

Supplementary Information

Supplementary Information

## Figures and Tables

**Table 1:** Crystal data and structure refinement for *M*La_10_Ir_4 _O_24_ (*M* = Sr, Ba) at 293(2) K.

Empirical formula	SrLa_10_Ir_4_O_24_	BaLa_10_Ir_4_O_24_
Formula weight	2629.52	2679.24
Temperature	293(2) K	293(2) K
Wavelength	0.71073Å	0.71073 Å
Crystal system	Tetragonal	Tetragonal
Space group	*I*4_1_/*a*	*I*4_1_/*a*
Unit cell dimensions	*a* = 11.5899(16) Å, α = 90.00° *b* = 11.5899(16) Å, β = 90.00° *c* = 16.255(3) Å, γ = 90.00°	*a* = 11.6569(6) Å, α = 90.00° *b* = 11.6569(6) Å, β = 90.00°*c* = 16.1673(9) Å, γ = 90.00°
Volume	2183.4(6) Å^3^	2196.9(2) Å^3^
Z	4	4
Density (calculated)	7.999 g/cm^3^	8.101 g/cm^3^
Absorption coefficient	45.869 mm^−1^	44.942 mm^−1^
F(000)	4432	4504
Crystal size	0.134 × 0.105 × 0.092 mm^3^	0.0237 × 0.0170 × 0.0084 mm^3^
θ range for data collection	3.52 to 24.98°	3.53 to 29.25°
Index ranges	−13<=h<=13, −13<=k<=12, −19<=l<=19	−15<=h<=15, −15<=k<=6, −22<=l<=22
Reflections collected	6661	5180
Independent reflections	968 [*R*_int_ = 0.1041]	1424 [*R*_int_ = 0.0754]
Completeness to θ = 29.25°	99.9%	95.6%
Refinement method	Full-matrix least-squares on F^2^	Full-matrix least-squares on F^2^
Data / restraints / parameters	968 / 0 / 94	1424 / 0 / 93
Goodness-of-fit	1.088	1.076
Final *R* indices [>2σ(I)]	*R*_obs_ = 0.0480, w*R*_obs_ = 0.1353	*R*_obs_ = 0.0532, w*R*_obs_ = 0.1219
*R* indices [all data]	*R*_all_ = 0.0563, w*R*_all_ = 0.1613	R_all_ = 0.0603, w*R*_all_ = 0.1268
Largest diff. peak and hole	4.662 and −2.694 e·Å^−3^	2.537 and −2.193 e·Å^−3^

**Table 2:** Atomic coordinates and equivalent isotropic displacement parameters (Å^2^ × 10^3^) for *M*La_10_Ir_4_O_24_ (*M* = Sr, Ba) at 293(2) K with estimated standard deviations in parentheses.

Label	*x*	*y*	*z*	Occupancy	U_eq_*
**SrLa**_**10**_**Ir**_**4**_**O**_**24**_					
Ir(1)	0.5000	0.5000	0	1	35(1)
Ir(2)	0	0.5000	0	1	35(1)
La(1)	0.7715(2)	0.4530(2)	0.1159(1)	1	38(1)
La(2)	0.7059(2)	0.7264(2)	0.348(1)	1	39(1)
La(3)	0.5000	0.7500	−0.1400(2)	1	52(1)
Sr(1)	0	0.7500	−0.1250	1	90(3)
O(1)	1.0087(12)	0.5358(15)	−0.1220(11)	1	44(4)
O(2)	0.8684(15)	0.6115(15)	0.0047(10)	1	46(4)
O(3)	0.3927(13)	0.4232(13)	0.0761(10)	1	44(3)
O(4)	0.5865(12)	0.5801(14)	0.0924(10)	1	42(3)
O(5)	0.6009(13)	0.3656(12)	0.0295(10)	1	40(3)
O(6)	1.1266(14)	0.6231(15)	0.0203(10)	1	42(3)
**BaLa**_**10**_**Ir**_**4**_**O**_**24**_					
Ir(1)	0.5000	0.5000	0	1	16(1)
Ir(2)	0	0.5000	0	1	16(1)
La(1)	0.2307(1)	0.4543(1)	0.1156(1)	1	17(1)
La(2)	0.2942(1)	0.7263(1)	0.0341(1)	1	19(1)
La(3)	0.5000	0.7500	−0.1416(1)	1	21(1)
Ba(1)	0	0.2500	0.1250	1	22(1)
O(1)	−0.0090(11)	0.5176(11)	−0.1233(7)	1	20(2)
O(2)	0.6096(11)	0.4306(12)	0.0793(7)	1	22(3)
O(3)	0.4080(12)	0.5748(11)	0.0933(8)	1	23(3)
O(4)	0.1339(12)	0.6108(12)	0.0011(7)	1	20(2)
O(5)	0.3963(12)	0.3661(12)	0.0272(9)	1	26(3)
O(6)	−0.1304(13)	0.6204(12)	0.0165(8)	1	24(3)

^*^U_eq_ is defined as one third of the trace of the orthogonalized U_ij_ tensor.

**Table 3:** Selected bond lengths [Å] for *M*La_10_Ir_4_O_24_ (*M* = Sr, Ba) at 293(2) K with estimated standard deviations in parentheses.

SrLa_10_Ir_4_O_24_		BaLa_10_Ir_4_O_24_	
Ir(1)-O(3)	1.967(15) × 2	Ir(1)-O(2)	1.983(12) × 2
Ir(1)-O(5)	2.006(14) × 2	Ir(1)-O(5)	2.023(13) × 2
Ir(1)-O(4)	2.030(16) × 2	Ir(1)-O(3)	2.046(12) × 2
Ir(2)-O(2)	2.001(16) × 2	Ir(2)-O(1)	2.006(12) × 2
Ir(2)-O(1)	2.028(17) × 2	Ir(2)-O(4)	2.026(13) × 2
Ir(2)-O(6)	2.073(16) × 2	Ir(2)-O(6)	2.086(13) × 2
