# Supplementary material for: New Insulating Antiferromagnetic Quaternary Iridates MLa10Ir4O24 (M = Sr, Ba)
Source: Sci Rep. 2015 Jul 1;5:11705. doi: 10.1038/srep11705 (PMC4486976; doi:10.1038/srep11705)
Supplement: Supplementary Information [file srep11705-s1.doc]

**New Insulating Antiferromagnetic Quaternary Iridates *M*La_10_Ir_4_O_24_ (*M* = Sr, Ba)**

Qingbiao Zhao,^1^*† Fei Han,^1^ Constantinos C. Stoumpos,^1^ Tian-Heng Han,^1,2^ Hao Li,^1^ and J. F. Mitchell^1^

*1. Materials Science Division, Argonne National Laboratory, Argonne, Illinois 60439, United States*

*2.* *Department of Physics, University of Chicago, Chicago, IL 60637, USA*

**Author information**

Corresponding author:

* Phone: (+86) 52416360 Fax: (+86) 52413903 Email: qbzustc@gmail.com

Current affiliation:

† *Shanghai Institute of Ceramics, Chinese Academy of Sciences, Shanghai, 200033, China*

**Table S1.** Anisotropic displacement parameters (Å^2^x10^3^) for *M*La_10_Ir_4_O_24_ (*M* = Sr, Ba) at 293(2) K with estimated standard deviation in parentheses.

| Label | U_11_ | U_22_ | U_33_ | U_12_ | U_13_ | U_23_ |
| --- | --- | --- | --- | --- | --- | --- |
| **SrLa_10_Ir_4_O_24_** |  |  |  |  |  |  |
| Ir(1) | 32(1) | 33(1) | 42(1) | 0(1) | 1(1) | 0(1) |
| Ir(2) | 31(1) | 33(1) | 41(1) | 0(1) | 0(1) | 0(1) |
| La(1) | 34(1) | 34(1) | 45(1) | 0(1) | 0(1) | 0(1) |
| La(2) | 36(1) | 34(1) | 48(1) | 2(1) | 0(1) | 0(1) |
| La(3) | 49(2) | 46(2) | 61(2) | -3(1) | 0 | 0 |
| Sr(1) | 29(2) | 29(2) | 213(9) | 0 | 0 | 0 |
| O(1) | 30(8) | 57(10) | 46(8) | -4(7) | 1(6) | 2(8) |
| O(2) | 39(9) | 43(9) | 54(10) | 13(7) | -15(7) | -6(7) |
| O(3) | 42(8) | 38(8) | 51(9) | -3(7) | -1(7) | 6(7) |
| O(4) | 27(7) | 47(8) | 52(9) | -1(6) | 3(6) | 3(7) |
| O(5) | 36(8) | 31(8) | 53(9) | 4(7) | 1(7) | -3(7) |
| O(6) | 37(8) | 46(9) | 42(8) | -2(7) | -3(7) | -6(7) |
| **BaLa_10_Ir_4_O_24_** |  |  |  |  |  |  |
| Ir(1) | 16(1) | 18(1) | 16(1) | 0(1) | 0(1) | 0(1) |
| Ir(2) | 16(1) | 16(1) | 15(1) | 0(1) | 0(1) | 1(1) |
| La(1) | 19(1) | 17(1) | 17(1) | 0(1) | 0(1) | 0(1) |
| La(2) | 19(1) | 18(1) | 19(1) | -2(1) | 0(1) | 1(1) |
| La(3) | 24(1) | 20(1) | 18(1) | 2(1) | 0 | 0 |
| Ba(1) | 20(1) | 20(1) | 27(1) | 0 | 0 | 0 |
| O(1) | 27(6) | 26(6) | 8(4) | -1(5) | -3(4) | -6(4) |
| O(2) | 20(6) | 30(7) | 17(5) | 6(5) | -7(4) | -6(5) |
| O(3) | 33(7) | 17(6) | 18(5) | 1(5) | 8(5) | -9(4) |
| O(4) | 21(6) | 22(6) | 16(5) | -3(5) | -3(4) | 1(4) |
| O(5) | 26(7) | 18(6) | 33(7) | -9(5) | 6(6) | -1(5) |
| O(6) | 27(7) | 24(6) | 21(5) | 11(5) | 2(5) | -2(5) |

The anisotropic displacement factor exponent takes the form: -2π^2^[h^2^a^*2^U_11_ + ... + 2hka^*^b^*^U_12_].
